# An appearance quality classification method for *Auricularia auricula* based on deep learning

**DOI:** 10.1038/s41598-023-50739-4

**Published:** 2024-07-05

**Authors:** Yang Li, Jiajun Hu, Haiyun Wu, Yong Wei, Huiyong Shan, Xin Song, Xiuping Hua, Wei Xu, Yongcheng Jiang

**Affiliations:** 1 College of Engineering and Technology, Tianjin Agricultural University, Tianjin, 300392 China; 2 Key Laboratory of Smart Breeding (Co-construction by Ministry and Province), Ministry of Agriculture and Rural Affairs (TJAU), Tianjin, 300392 China; 3 College of Mechanical Engineering, Jiamusi University, Jiamusi, 154007 China

**Keywords:** Engineering, Electrical and electronic engineering, Mechanical engineering

## Abstract

The intelligent appearance quality classification method for *Auricularia auricula* is of great significance to promote this industry. This paper proposes an appearance quality classification method for *Auricularia auricula* based on the improved Faster Region-based Convolutional Neural Networks (improved Faster RCNN) framework. The original Faster RCNN is improved by establishing a multiscale feature fusion detection model to improve the accuracy and real-time performance of the model. The multiscale feature fusion detection model makes full use of shallow feature information to complete target detection. It fuses shallow features with rich detailed information with deep features rich in strong semantic information. Since the fusion algorithm directly uses the existing information of the feature extraction network, there is no additional calculation. The fused features contain more original detailed feature information. Therefore, the improved Faster RCNN can improve the final detection rate without sacrificing speed. By comparing with the original Faster RCNN model, the mean average precision (mAP) of the improved Faster RCNN is increased by 2.13%. The average precision (AP) of the first-level *Auricularia auricula* is almost unchanged at a high level. The AP of the second-level *Auricularia auricula* is increased by nearly 5%. And the third-level *Auricularia auricula* AP is increased by 1%. The improved Faster RCNN improves the frames per second from 6.81 of the original Faster RCNN to 13.5. Meanwhile, the influence of complex environment and image resolution on the *Auricularia auricula* detection is explored.

## Introduction

*Auricularia auricula* is a large edible fungus used both as medicine and food with high health value^1–4^. The rapid development of the *Auricularia auricula* industry greatly promotes its export and sales. The production and processing industries of *Auricularia auricula* have a positive role in promoting economic efficiency, which is of great significance to the development of forest economy and the increase of farmers’ income. In addition to the conventional features such as color, hue, shape, and size, *Auricularia auricula* exhibits other features as wrinkles, diseases, and damage, which directly impact its appearance quality and result in undesireable taste. Describing and quantifying these features accurately is challenging, and currently, only experienced workers can achieve reliable appearance quality classifiation outcomes. China is the world’s largest Auricularia auricula producer, accounting for more than 90% of the world’s output. In 2018, two adjacent provinces, Heilongjiang Province and Jilin Province, produced 476,000 tons of *Auricularia auricula*, accounting for 70.6% of the national total^5^. The planting area is extensive, but the key planting area is relatively concentrated, which has led to few studies on *Auricularia auricula*. As far as we know, there have been no studies on the appearance quality of *Auricularia auricula*.

Therefore, *Auricularia auricula* with different appearance qualities needs to be graded manually. At present, it is mainly done by manpower which is labor-consuming and inefficient. Due to the contingency, misjudgment, and discontinuity of manual grading, it is of practical significance to realize the automatic appearance quality classification of *Auricularia auricula*. In this way, we can also improve the classification consistency and accuracy, while reduce labor costs. After years of efforts by researchers, deep learning has made significant progress and applications in many fields^6,7^. In view of the superior performance of deep learning methods in image recognition and other fields^8,9^, it should also have good application value in the appearance quality classification of black fungus, and can effectively solve the core problems of machine vision classification of *Auricularia auricula*, and greatly improve the classification quality. However, to our knowledge, the appearance feature attributes of *Auricularia auricula* has not yet been studied from the perspective of machine vision, especially through deep learning methods. By making full use of shallow feature information to realize target detection, this paper tries to establish a multiscale feature fusion detection model based on Faster RCNN, which is called improved Faster RCNN, to fulfill the automatic classification of *Auricularia auricula* based on their appearance quality.

Target detection is an important research part of current machine vision, and how to improve its accuracy and speed is the current research focus^10^. The faster regional-based convolutional neural network (Faster RCNN) algorithm is a result of merging region proposal network (RPN) and Fast-RCNN algorithms into a single network. And it has good qualities in terms of accuracy and speed. Therefore, the algorithm has a large number of applications. Wan and Goudos applied Faster RCNN directly for multi-class fruit detection using a robotic vision system^11^. They found that the system achieved higher detecting accuracy and lower processing time than the traditional detectors. For detection and classification applications, various improvements to Faster RCNN have been proposed. Faster RCNN was improved with a deep reinforcement learning model and was used for intelligent video anomaly detection and classification^12^. By using high-resolution network as the backbone network, Faster RCNN was improved to detect hydroponic lettuce seedlings’ status^13^. By adversarial occlusion network, the Faster RCNN was improved to utilize in underwater target detection, and the increase of mAP is 2.6% compared with the standard Fater RCNN network^14^. However, Faster RCNN has not been used in detecting, grading, and classifying *Auricularia auricula*.

The research on quality evaluation of *Auricularia auricula* mostly focused on the study of quality components such as total saccharide content^15^, amino acids^16^, and volatile components^17^. And the evaluation methods were mainly based on electronic tongue and nose^17^, near-infrared technologies^15^, and acid hydrolysis^16^. However, there was no research on evaluating and classifying the appearance quality of *Auricularia auricula* by machine vision. To make this deep learning technology more suitable for the specific field of appearance quality evaluation of *Auricularia auricula*, we proposed a multiscale feature fusion detection model to improve the standard Faster RCNN.

In this study, 2000 dried *Auricularia auricula* of three classes are graded according to the national standard of appearance quality, and 6000 images of *Auricularia auricula* samples from 3 different perspectives are collected. To improve the quality classifying accuracy and real-time performance, an appearance quality classification method for *Auricularia auricula* is constructed based on an improved Faster RCNN framework. The improved Faster RCNN method is compared with the other 4 different algorithms. At the same time, the influence of complex conditions and image resolution on the *Auricularia auricula* detection is also explored. And some suggestions and methods are finally proposed to reduce these possible negative effects.

## Materials and methods

### Experimental data

#### Data collection

For data acquisition, the primary equipment was the Huawei Honor V30 Pro Android smartphone, which employed automatic white balance and optical focus settings to capture images of *Auricularia auricula*. The data acquisition platform for *Auricularia auricula* image data included a tripod to stabilize the camera and a bracket to position the lighting lamp and relevant accessories. These devices set up on a horizontal tabletop. Throughout the data collection process, the camera lens maintained a distance of 50 cm from the tabletop. The lighting was a ring LED light specifically designed for machine vision applications. Under vertical illumination conditions, the light was fixed on the tripod, and the camera lens was positioned directly above the central hollow area of the LED ring light. When capturing images of *Auricularia auricula* illuminated obliquely, the light was placed on the light bracket near the camera. By adjusting the height, angle, and color of the light on the bracket, *Auricularia auricula* image data under various complex lighting conditions could be obtained. During the photography process, a sheet of A4 paper was placed directly beneath the camera lens on the tabletop, and the classified *Auricularia auricula* samples were individually placed on the paper for image capture.

2000 dried *Auricularia auricula* of three classes, which were graded according to the national standard of appearance quality of dried Auricula auricula products GB/T 6192-2019^18^, were selected as the experimental materials in this paper.

**Figure 1:**
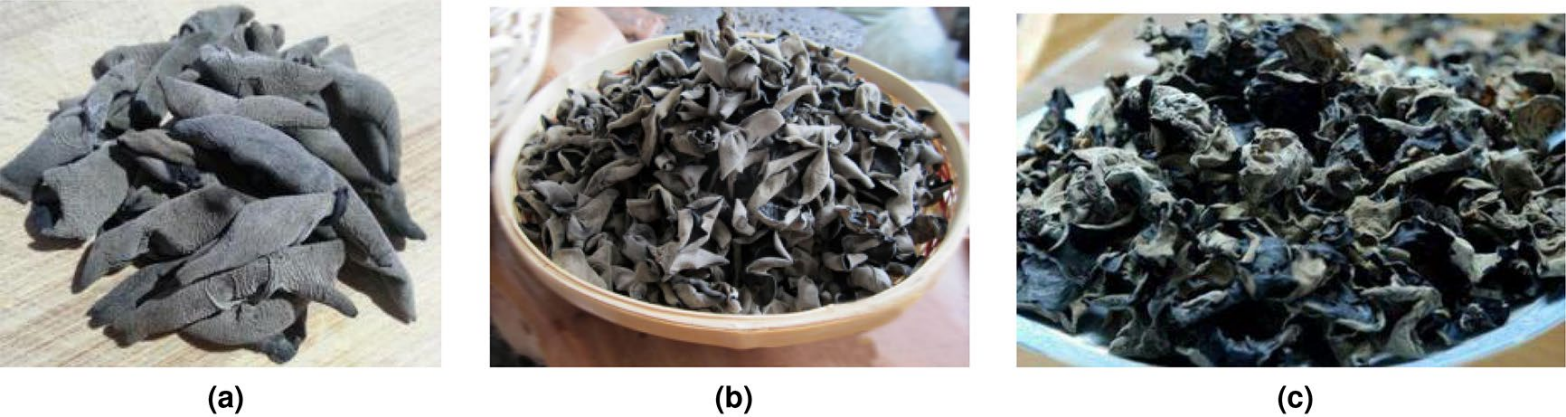
Three classes of dried *Auricularia auricula* products graded based on GB/T 6192-2019^18^. (**a**) Graded as the 1st-level, (**b**) the 2nd-level, and (**c**) the 3rd-level.

Following the standard of image collection, images of each *Auricularia auricula* sample were collected from the front, back, and side perspectives. Finally, 6000 images of *Auricularia auricula* samples from the three different perspectives were collected. This dataset has been made publicly available at https://github.com/liyang005/Auricularia_auricula-dateset. Because of the too large original image size of $2736\times 2648$ pixels, the image size is uniformly adjusted to the size of $800\times 600$ to facilitate our model training. Figure 1 shows three classes of dried *Auricularia auricula* products which were graded by GB/T 6192-2019^18^.

#### Image labeling

**Figure 2:**
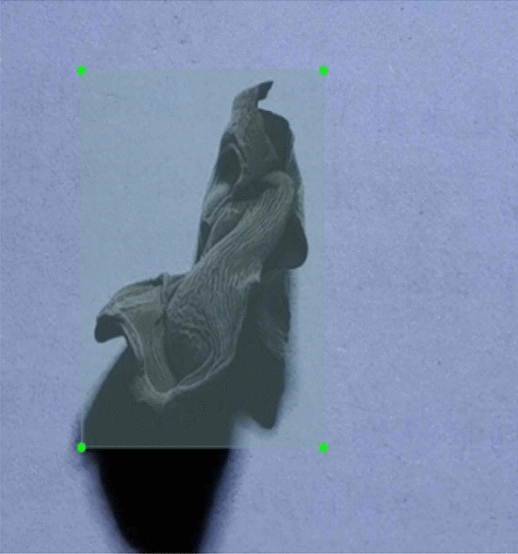
Schematic diagram of *Auricularia auricula* labeling. Framing the *Auricularia auricula* by a rectangular box.

In this study, the collected images were labeled, as depicted in Fig. 2. By framing the *Auricularia auricula* in each image, its corresponding coordinates were obtained, representing the upper left corner and the lower right corner of the rectangular box enclosing the *Auricularia auricula*. The labeling process followed the VOC^19^ dataset format and involved creating three folders: JPEGImages, Annotations, and ImageSets.

**Figure 3:**
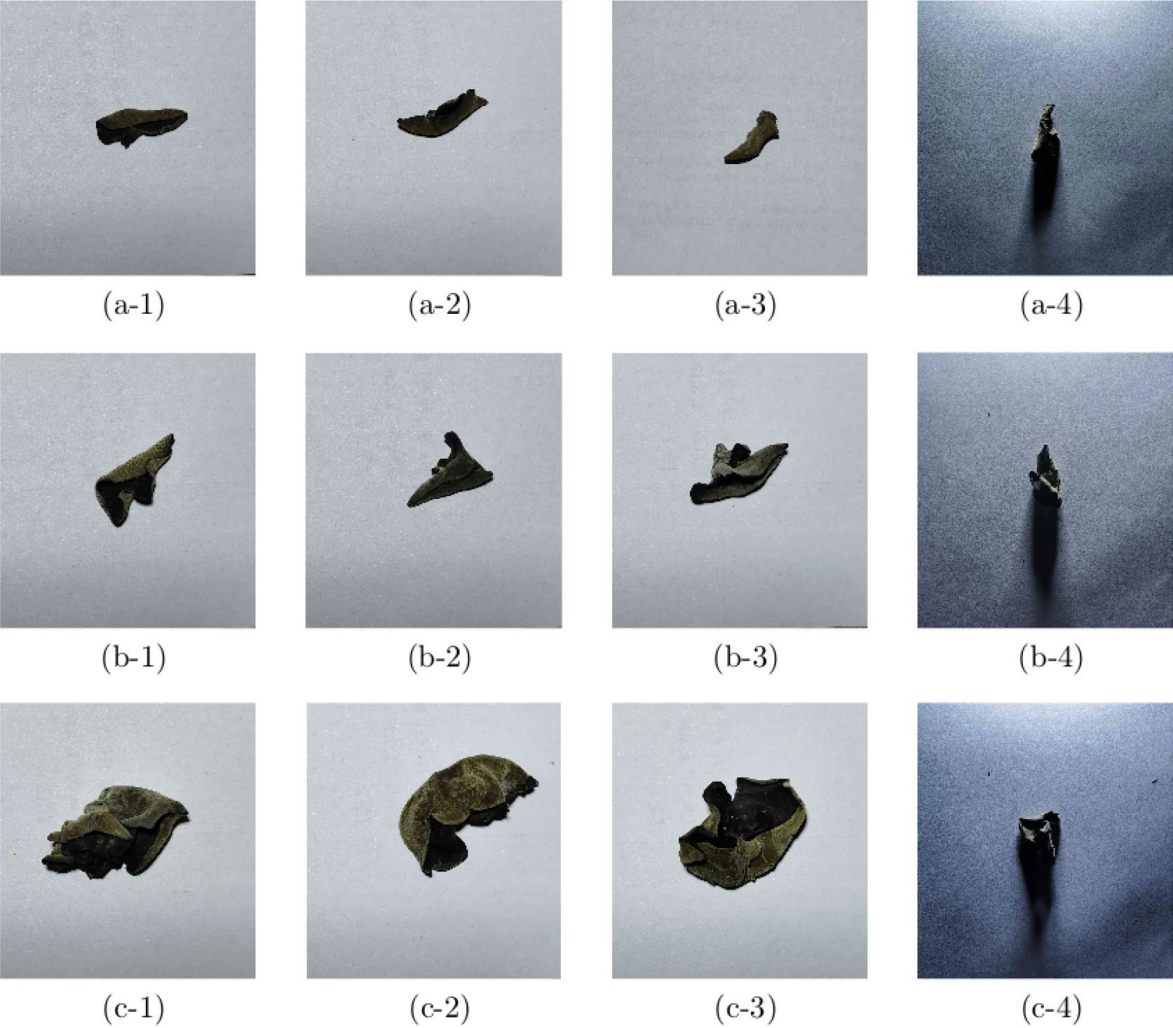
*Auricularia auricula* data set.

The JPEGImages folder was used to store the collected images, including training and test images. These images were numbered. The *Auricularia auricula*r’s appearance quality of each image was labeled by LABELIMG^20^. The default VOC XML format was used for creating labels, and the labeling result of each image was stored in its corresponding XML file. The Annotations folder was used to store these XML files, which contained labeling information corresponding to the original image. TXT files in the ImageSets folder specified whether the image data belonged to a training or validation set. The ratio of the training set and the test set was 8:2. Finally, the appearance quality data set of *Auricularia auricula* was obtained. Figure 3 shows the data set of appearance quality of *Auricularia auricula* established in this paper.

### Methodology

#### Overview of Faster RCNN

**Figure 4:**
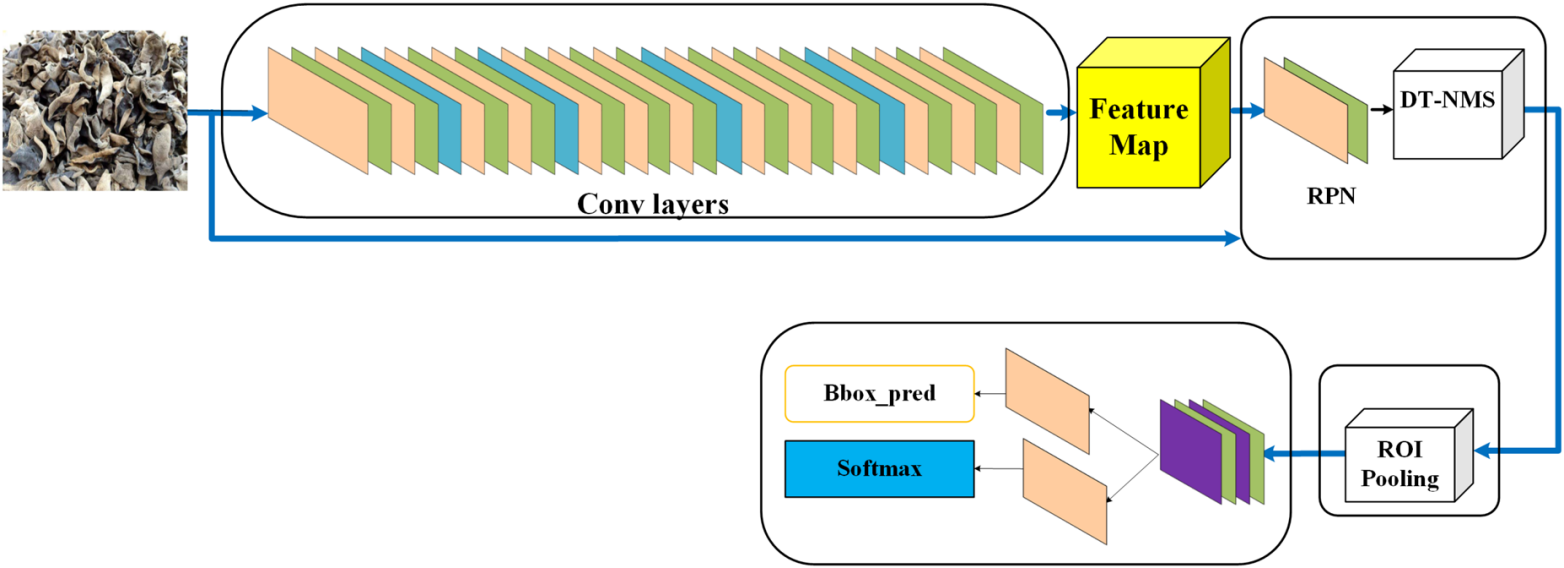
Faster RCNN.

This article used an improved Faster RCNN^21^ (Fig. 4) to train the model. Firstly, the Faster RCNN was reviewed.

The Faster RCNN algorithm can be understood in two parts: Region-proposal Network (RPN)^22^ and Fast RCNN^23^. Faster RCNN replaces the Selective Search (SS) algorithm^24^ in Fast RCNN by RPN. The Conv layers represent the feature extraction convolutional neural network, and Feature Map is the corresponding feature obtained in Fig. 4. RPN is the Region-proposal Network, and is mainly used to generate the Region Proposal box. Its details will be introduced in the next subsection. DT-NMS is the dual threshold-non-maximum suppression algorithm, which is designed to solve the problem of target missed detection and target duplicate detection in the single-threshold NMS algorithm. ROI pooling is represented the region of interest pooling layer, which takes the output of the convolutional neural network and the suggested region as input, and outputs the features extracted from each suggested region. Bbox_pred is the bounding box prediction of each suggested region in the classification and regression section of the Faster RCNN. And Softmax is the softmax regression used to classify the category of the input fungus image. For each *Auricularia auricula* image inputted into Faster RCNN, the Bbox_pred gives where the *Auricularia auricula* is in the image by a box and the Softmax gives which category the *Auricularia auricula* is. And the Faster RCNN algorithm flow can be divided into three steps:

*Step 1:* Input the image into the feature extraction network to obtain the corresponding feature.

*Step 2:* Use the RPN structure to generate the candidate frame, and project the candidate frame generated by the RPN onto the feature map to obtain the corresponding feature matrix.

*Step 3:* Scale each feature matrix to a $7\times 7$ size feature map through the ROI pooling^25^ layer, and obtain the prediction probability and bounding box regression parameters by flattening a series of fully connected layers.

Region-proposal Network (RPN) is in the middle part of the whole Faster RCNN structure, and its main function is to generate the Region Proposal box. RPN adds a $3\times 3$ sliding window to the last layer of VGG16^26^. Each sliding window is initialized as a reference area with an Anchors center. As long as the coordinates of the sliding window point are known, the specific coordinates of each Anchor box^27^ can be calculated. Finally, the classification function (Softmax) is used to generate the foreground background classification and prediction box, and the ROI Pooling is applied to the same size for subsequent processing.

Faster RCNN realizes the end-to-end training process, combining Region proposal, Feature extraction, target Classification, and Bounding-box regression. The four processes are all integrated into the CNN network. Compared with its previous two generations of algorithms, Fast RCNN is divided into two parts. One part is named Region proposal, in which the SS algorithm is used to generate 1000–2000 region proposals for each figure. This part is not included in the CNN network. The other part is the CNN network, which consists of the remaining algorithms of the Fast RCNN. In RCNN, the SS algorithm is used for region proposal, feature extraction is obtained by a separate CNN network, the classifier is a separately trained SVM classifier, and boundary box regression parameters are obtained by a separately trained boundary box regressors. Therefore, the framework from RCNN to Fast RCNN to Faster RCNN is more and more concise.

#### Fast RCNN

Fast RCNN uses the entire image as input to the convolutional neural network to extract features instead of a single suggested region. In addition, the network usually involves testing or updating model parameters.

Suppose the input is an image, and the output form of the convolutional neural network is $1\times c\times \text {h}_1\times \text {w}_1$. The selective search generates a total of n suggested regions. In the output of the convolutional neural network, the region of interest is marked with the proposed region. To output after concatenation, these regions of interest must be extracted with the same form of features (assuming that the height $\text {h}_2$, and the width $\text {w}_2$). Fast RCNN introduces the region of interest pooling (ROI pooling) layer, which takes the output of the convolutional neural network and the suggested region as input, and outputs the features extracted from each suggested region with the shape of $\text {n}\times \text {c}\times \text {h}_2\times \text {w}_2$. The fully connected layer transforms the output to the shape $\text {n}\times \text {d}$, where the hyperparameter $\text {d}$ is determined by the model. The output of the fully connected layer is converted to $\text {n}\times \text {q}$ and is classified using softmax^28^ regression ($\text {q}$ is the number of categories). When predicting the bounding box, the output of the fully connected layer is converted to $\text {n}\times 4$ and predicts the class and bounding box of each suggested region.

#### The improved Faster RCNN by multi-scale feature fusion

**Figure 5:**
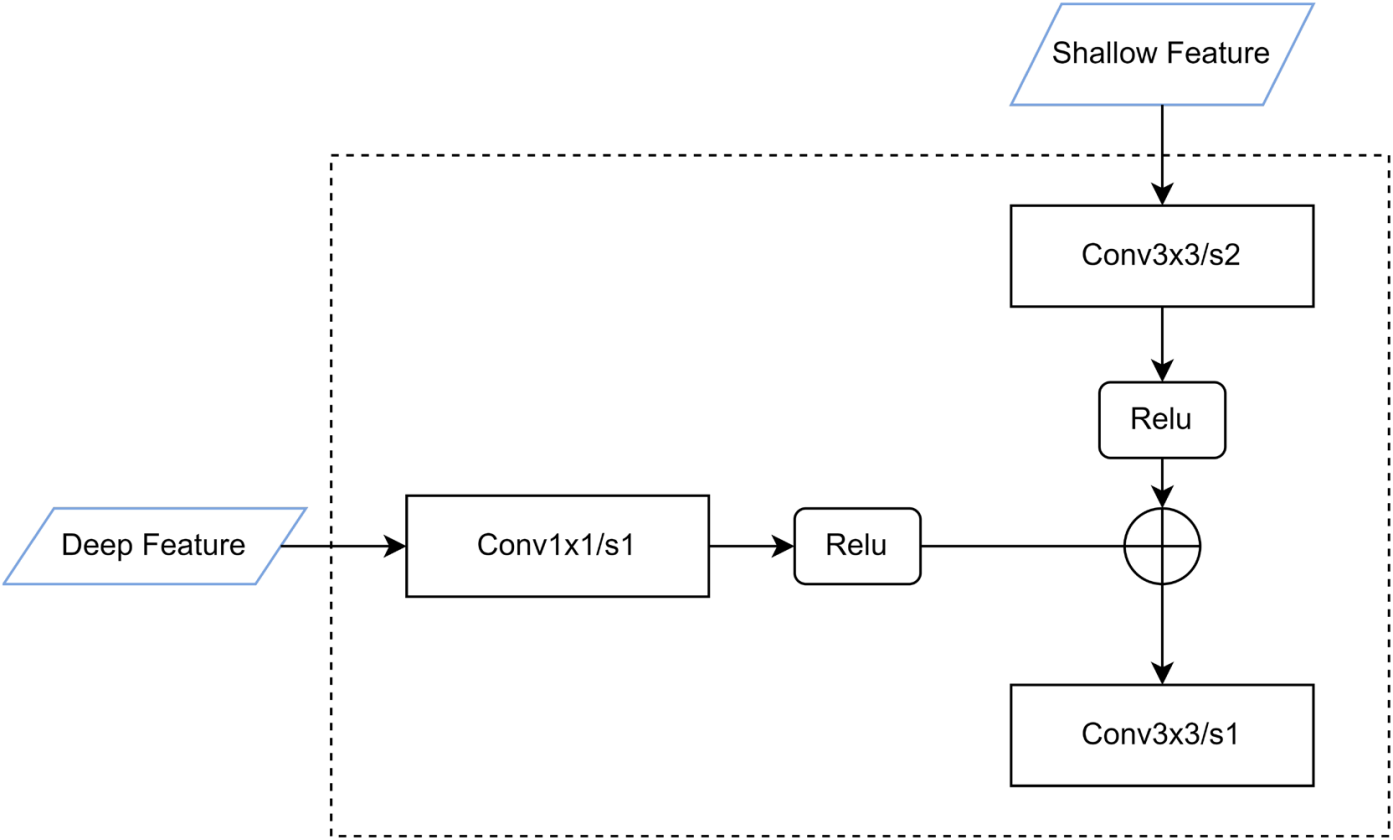
Schematic diagram of the fusion of two adjacent feature layers in a bottom-up approach.

**Figure 6:**
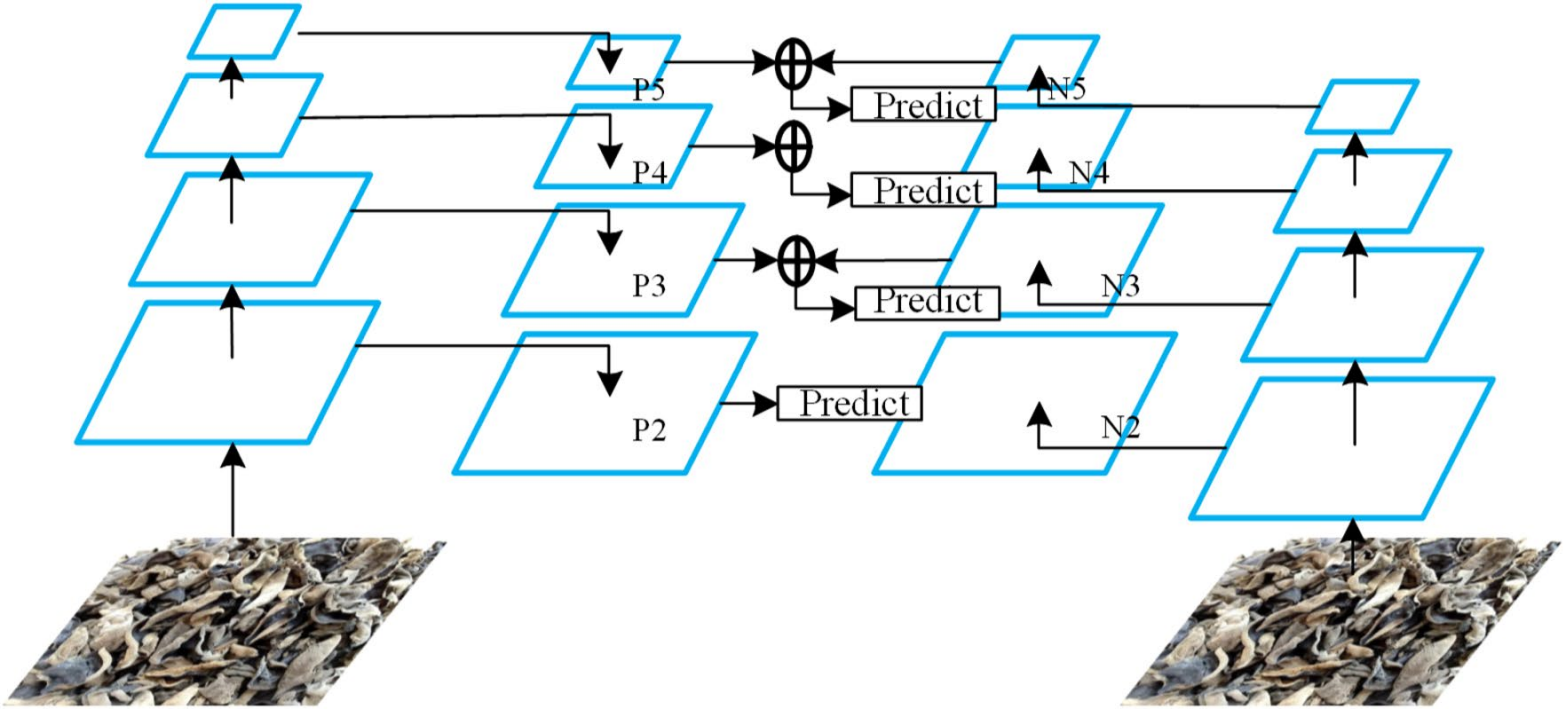
Feature fusion model with multi-scale detection function.

The bottom-up multi-layer and multi-scale feature fusion method were introduced, and the feature extraction structure of VGG16 was fused with the Feature Pyramid prediction structure of FPN^29^ to build a Feature fusion model with multi-scale detection. The experimental method was adopted to train and optimize the improved VGG16 network structure, and the number of channels in each convolutional layer was set to 256 to reduce the number of parameters and facilitate feature fusion. Figure 5 shows the diagram of the fusion of two adjacent feature layers in bottom-up mode, and Fig. 6 shows the feature fusion model with a multi-scale detection function.

Firstly, the underlying feature (the shallow feature in Fig. 5) passes through a convolution layer with a convolution stride of 2, and its convolution kernel size is 3$\times$3 (The Conv3$\times$3/s2 in Fig. 5), the function of this convolutional layer is to change the width and height of the input feature map to half of the original, after going through a non-convolutional layer (Relu); and the top-level feature goes through a convolution step The convolution layer (Cov1$\times$1/s1) with a length of 1 and a convolution kernel size of 1$\times$1, the function of this convolution layer is to reduce the number of output feature channels and then go through a nonlinear layer (Relu); finally, the above two results are summed element by element, and then go through a convolution layer with a convolution stride of 1 and a convolution core size of 3 $\times$ 3. This convolution layer is used to remove feature aliasing and obtain new fusion features. The number of channels in all convolutional layers in Fig. 5 is 256, which is to reduce the number of parameters and facilitate feature fusion. During training, the backbone network adopts the modified VGG-16.

The boxes ($\square$) in Fig. 6 represent element-wise summation. The left half of the figure is the original FPN network, where P2 to P5 represent 4 multi-scale features after fusion, and the width or height of adjacent feature layers differ by 2 times. P5 is directly copied from the last layer of the backbone network, which is rich in semantic information. But P5 does not integrate the underlying features and lacks details such as target location information and context information. For this reason, this paper introduces a Bottom-Up multi-layer feature fusion method based on FPN, which is the right half of Fig. 6. N2 to N5 represents the features fused from the bottom to the top. The height or width of the two adjacent feature layers from N2 to N5 are twice different. N2 is copied from the convolutional layer in the backbone network. The specific fusion method of the fusion feature layer is shown in Fig. 5.

Formula (1) is the feature mapping based on ROI size selection.

1 $$\begin{aligned} k = \lfloor k_0+\log _2\left( \sqrt{\text {wh}}/I\right) \rfloor \end{aligned}$$

$\lfloor x \rfloor$, denoted the floor function of a real number *x*, is defined to be the greatest integer that is less than or equal to *x*. *I* is the size of the pre-training graph, $k_0$ is the reference value (the level of the pre-training ROI), representing the output of layers, and $\text {w}$ and $\text {h}$ are the width and height of ROI.

#### Scores for the assessment of the performances of learning models

To evaluate object detection models used in this paper, the average precision (AP)^30^ was used, which involves the following definitions.

True positive (TP) is a given *Auricularia auricula*’s grade being correctly identified as its labeled grade. Precision represents how much of the target detected by the model is the real target object; Recall represents the proportion of all real targets detected by the model. AP is determined by precision and recall^30^.

Intersection over Union (IoU)^31^ is the overlap of the two regions occupying the total area of the two regions, which measures the degree of overlap of these regions.

The object detection result was divided into four situations by the relationship between the value of IoU and the confidence threshold, which are shown in Table 1. True is defined by the IoU value of a target box in the data set greater than 0.5. False is defined by the IoU value of all target boxes in the data set greater than 0.5. Positive is defined by the detected rectangular frame being greater than the confidence threshold. Negative is defined by the detected rectangular box being less than the confidence threshold. True positive (TP) is IoU greater than 0.5 and detected, true negative (TN) is IoU greater than 0.5 and not detected, false positive (FP) is a false target but the rectangular box score is greater than the confidence level, false negative (FN) is a false target and the rectangular box score is also less than the confidence level.

**Table 1:** Conceptual definition table of forecast results.

	Positives	Negatives
True	TP (true positives)	TN (true negatives)
False	FP (false positives)	FN (false negatives)

The precision in the precision–recall (P–R) curve can be calculated by formula (2),

2 $$\begin{aligned} \text {Precision}=\frac{\text {TP}}{\text {TP}+\text {FP}} \end{aligned}$$

The recall is shown in formula (3).

3 $$\begin{aligned} \text {Recall}=\frac{\text {TP}}{\text {TP}+\text {FN}} \end{aligned}$$

### Training platform

The improved Faster RCNN *Auricularia auricula* classification model was run on an AMD R5 3600 central processor (CPU) and NVIDIA GeForce RTX 2070 graphics card (GPU) equipped with Windows10 64-bit operating system. Memory was of dual channel 16 GB 3200 MHz. The test code was written in Python 3.5.6 and the code editor was Visual Studio Code. The deep learning framework used is TensorFlow-GPU 1.13.2, and the development environments of the GPU parallel computing framework were CUDA10.0 and CUDN7.4.1.5.

## Results

### Model training

To train the improved Faster RCNN model, these 6000 *Auricularia auricula* images were then divided into two parts, one was 4800 training sets, accounting for 80%, and the other was 1200 test sets, accounting for 20%. The training was carried out for 100 iterations steps, and each iteration step took about 0.3 seconds for a single sample. When the training reached 80 steps, the loss converged gradually and tended to 0.31; after 100 iteration steps of the model training, the loss was 0.306, as shown in Table 2.

**Table 2:** The loss value and training time of the ordinal number of iteration step.

The *n*th iteration step	Loss	Time (min)
80	0.314	13
85	0.318	15
90	0.314	12
95	0.313	17
100	0.306	14

**Figure 7:**
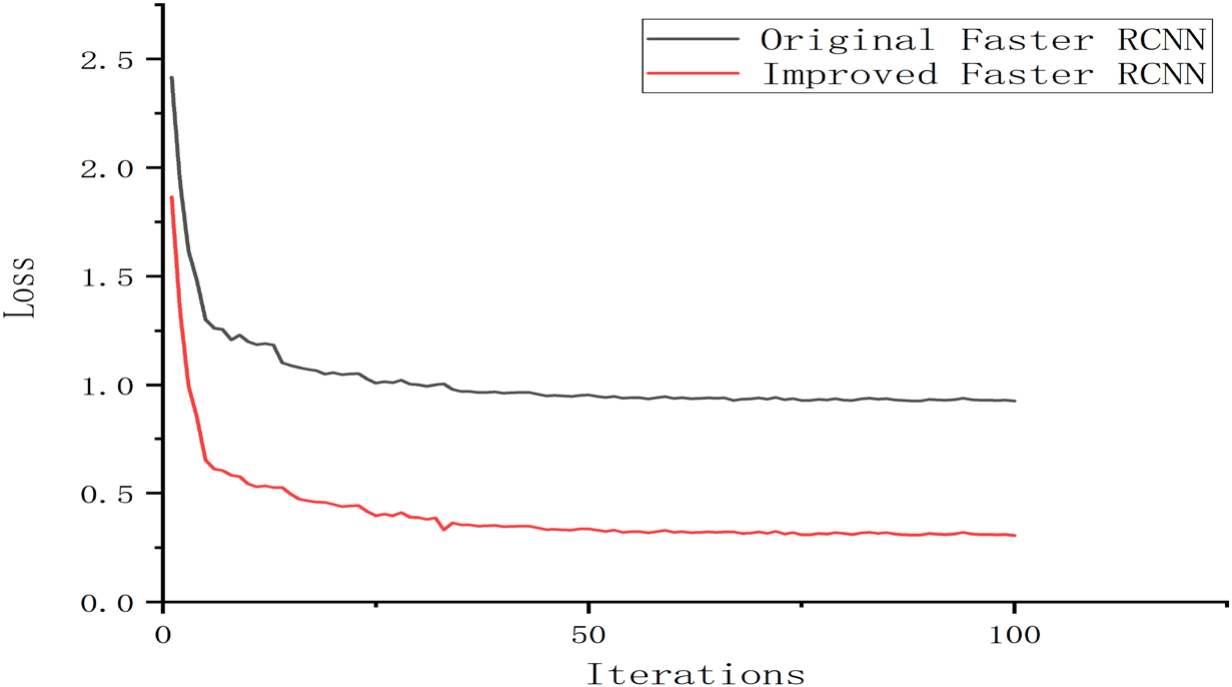
Comparison of the convergence of loss between improved Faster RCNN and original Faster RCNN.

After the training, all losses can be drawn, as shown in Fig. 7. It can be seen that after a long time of training, loss finally converges to 0.3, which was greatly improved compared with the original Faster RCNN.

### The influence of image resolution and complex environment on the models

**Figure 8:**
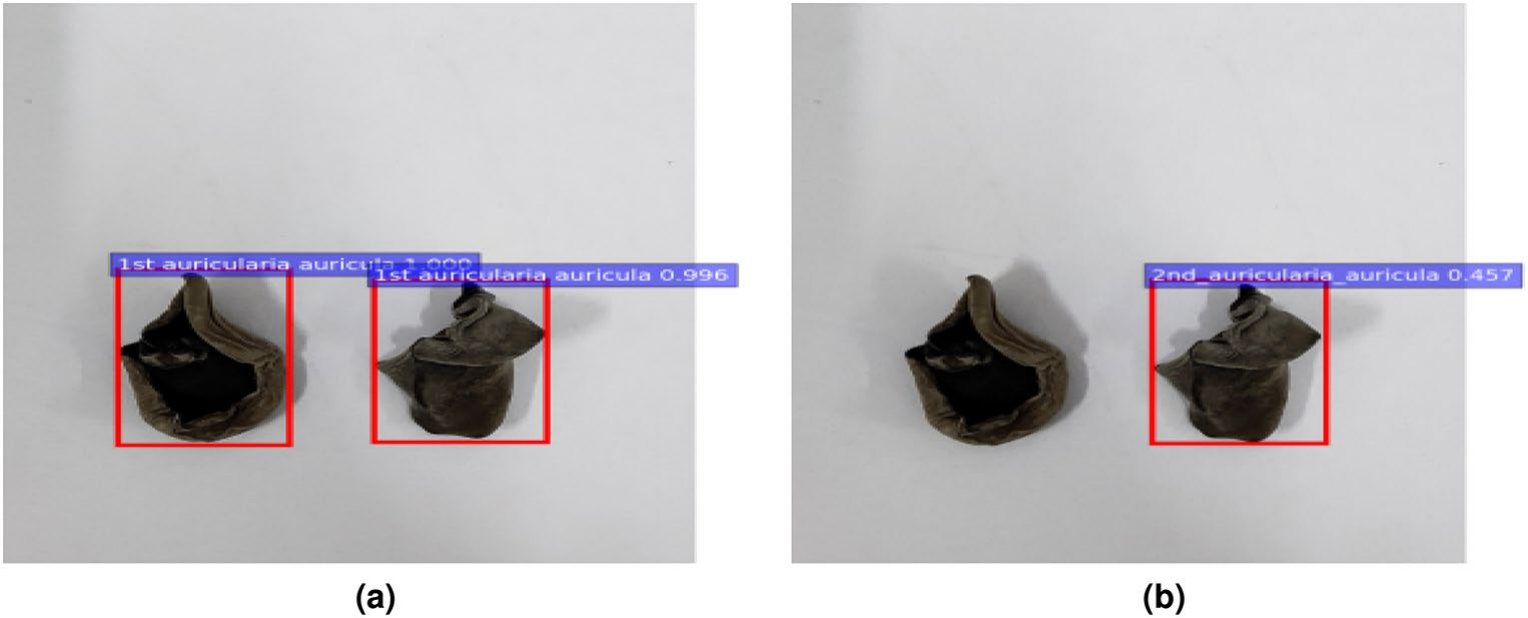
The appearance quality classifying results with different image sizes. (**a**) With $800\times 600$, (**b**) with $5000\times 3000$.

In this experiment, image sets of different sizes were used for network training to obtain a better detection effect for the *Auricularia auricula* classifying model. The size of images in Fig. 8a was $800\times 600$ pixels, while the size of images in Fig. 8b was $5000\times 3000$ pixels. It could be seen that the two *Auricularia auricula* in lower pixel images were successfully detected and classified with high scores and accurate location coordinates; All *Auricularia auricula* could not be successfully detected in the images with higher pixels, and even the one detected had a low score and could not be successfully classified. Therefore, the *Auricularia auricula* with $800\times 600$ pixel images had better classifying results.

**Figure 9:**
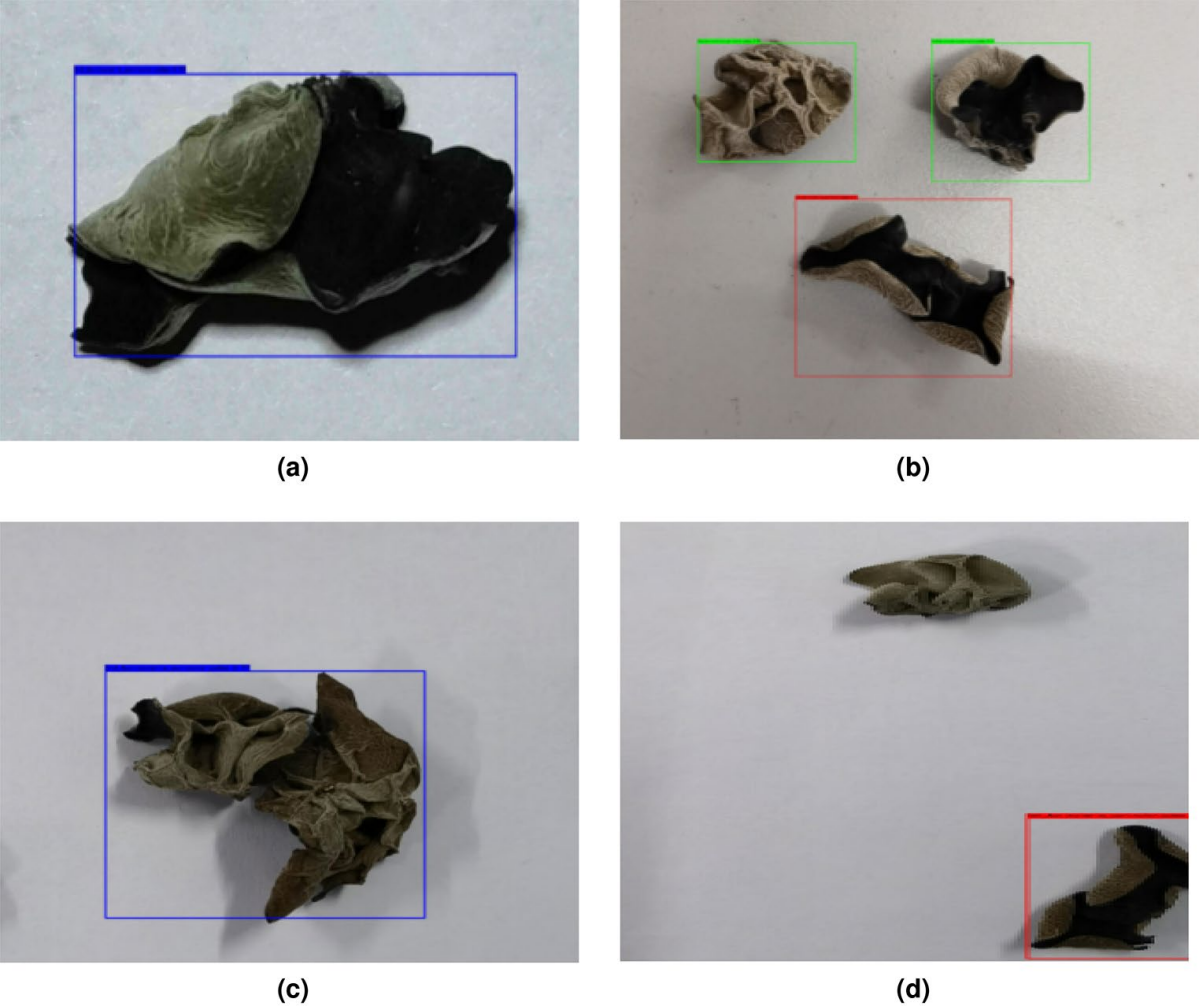
Classification results under complex environment. (**a**) With single *Auricularia auricula*, (**b**) with three separate *Auricularia auricula*, (**c**) with two *Auricularia auricula* stuck together, (**d**) with mutiple *Auricularia auricula* under complex lighting conditions.

When unlabeled *Auricularia auricula* images were input into the trained model for detection, the complex detection environment had an impact on black fungus detection, as shown in Fig. 9.

Complex environments, such as an image with many *Auricularia auricula*, an image with the *Auricularia auricula* getting stuck together, and images under complex lighting conditions, would adversely affect the classification results of the model. In the case of single *Auricularia auricula*, shown in Fig. 9a, the trained Faster RCNN *Auricularia auricula* classification model could correctly identify the object in the image, and classify it as the third-grade *Auricularia auricula* with a probability of 0.93; When there were three *Auricularia auricula* in an image, shown in Fig. 9b, the improved Faster RCNN classification model could correctly distinguish them, and give their corresponding appearance qualities and coordinates; Fig. 9c shows two black *Auricularia auricula* with slight adhesion. The Faster RCNN black auricula detection model could not correctly distinguish the two black *Auricularia auricula* in the picture but regarded it as one black auricula. When two *Auricularia auricula* got stuck together, the improved Faster RCNN classification model mistakenly identified them as one. For an image with multiple *Auricularia auricula* taken under complex lighting conditions, the classification model recognized and correctly classified the first-level *Auricularia auricula* in the lower right corner in Fig. 9d, and ignored all other *Auricularia auricula* in the image. In these complex environments, the performance of algorithms is not as good as in normal environment.

Therefore, in order to ensure the effective performance of the appearance quality classification model, it was crucial to establish an optimal classification environment. This involved classifying a single *Auricularia auricula* at a time and carefully selecting the appropriate lighting source. The choice of the lighting source played a significant role in enhancing the classification performance of the model.

To apply the appearance quality classification system of *Auricularia auricula*, the requirements of real-time classification should be fully met. The model’s real-time performance was described by the frame rate. The frame rate of images with different numbers of *Auricularia auricula* was analyzed. The analysis results are shown in Table 3.

**Table 3:** The frame rate of the improved and original Faster RCNN in real time under different number of *Auricularia auricula*.

Number *Auricularia auricula*	Improved (frames/s)	Original (frames/s)
None	15.37	8.76
One	13.19	6.81
Many	12.04	6.04

The results show that when there is no *Auricularia auricula* in the detection screen, the frame rate of image processing of the improved Faster RCNN reaches 15.37 frames per second. When one *Auricularia auricula* appears on the detection screen, the frame rate slows down to 13.19 frames per second. As the number of *Auricularia auricula* in the detection screen increases, the frame rate of image processing decreases to 12.04 frames/s.

Compared with the original model, the real-time performance of the improved model is greatly improved and can meet the actual production requirements.

### Comparison of different algorithms

To select the improved Faster RCNN as the appearance quality classification method for *Auricularia auricula*, it was compared with other algorithms, such as Single Shot MultiBox Detector (SSD), RCNN, Fast RCNN, and the Faster RCNN in this subsection.

**Figure 10:**
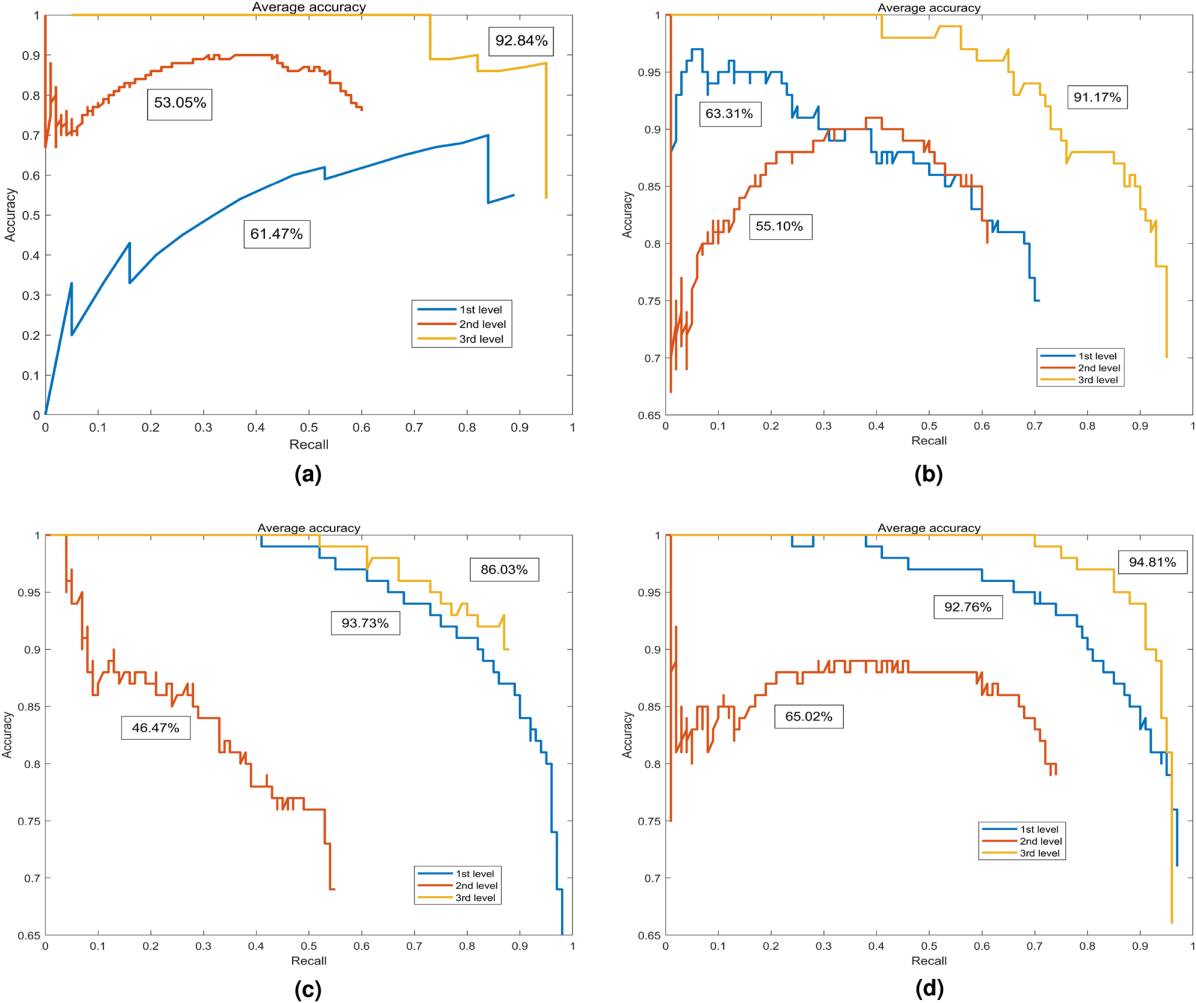
Average accuracy of the four algorithms. (**a**) The SSD model, (**b**) is of the RCNN model, (**c**) is of the Fast RCNN model, (**d**) is of the Faster RCNN model.

During the training, network parameters were adjusted in real-time according to the loss value and running time of each step.

**Figure 11:**
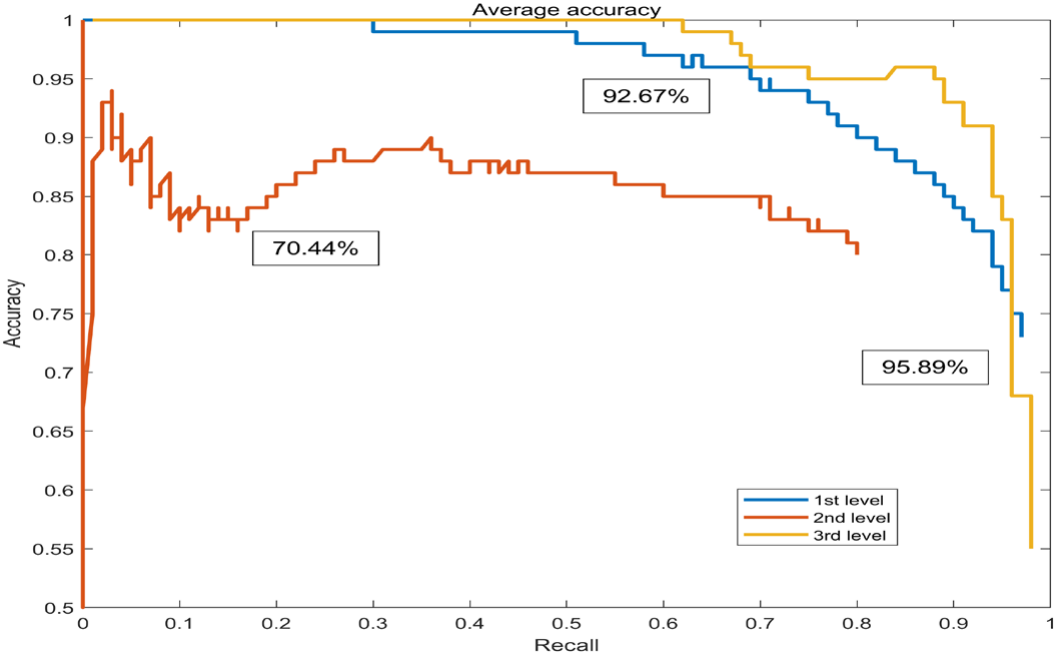
Average accuracy of the improved Faster RCNN.

The precision–recall (P–R) diagrams of the running results of each model are shown in Figs. 10 and 11. By comparing the improved Faster RCNN model, as shown in Fig. 11, with other models, shown in Fig. 10 from (a) to (d), it can be seen that the average accuracy rate has been improved, especially for the 2nd-level *Auricularia auricula*, and the AP has been improved greatly. Therefore, the improved Faster RCNN method has met the actual needs in terms of classification accuracy.

The loss, the mean average precision (mAP), and frames per second (FPS) of the five algorithms are listed in Table 4. In terms of model performance, the improved Faster RCNN has a lower loss than other algorithms, and its convergence effect is better than other algorithms. Therefore, the improved Faster RCNN has a significant performance improvement compared to other algorithms. In terms of detection performance, the mAP values of the improved Faster RCNN are higher than the other four models. This shows that its recall and precision, model detection bounding frame accuracy, and model classification effect are better. Compared with other algorithms, the improved Faster RCNN can meet the requirements of real-time detection.

By comparing the other four algorithms, the improved Faster RCNN contains more original detailed feature information by fusing shallow feature information. Meanwhile, the fusion algorithm directly uses the existing information of the feature extraction network. The improved Faster RCNN can improve the detection rage without sacrificing speed.

**Table 4:** Performances of different algorithms.

Algorithms	Loss	mAP (%)	FPS
SSD	2.4	69.12	46.32
RCNN	0.6	69.89	–
Fast RCNN	0.36	75.41	–
Faster RCNN	0.8	84.20	6.81
Improved Faster RCNN	0.3	86.33	13.19

In summary, by full use of shallow feature information, the original Faster RCNN is improved by establishing a multiscale feature fusion detection model to complete *Auricularia auricula* detection. Because this proposed algorithm directly fuses shallow features with rich detailed information with not additional operation, the improved Faster RCNN can improve the final detection rate without sacrificing speed. Therefore, compared with the original Faster RCNN model, the mean average precision (mAP) of the improved Faster RCNN is increased by 2.13%. The average precision (AP) of the first-level *Auricularia auricula* is almost unchanged at a high level. The AP of the second-level *Auricularia auricula* is increased by nearly 5%. And the third-level *Auricularia auricula* AP is increased by 1%. The improved Faster RCNN improves the frames per second from 6.81 of the original Faster RCNN to 13.5. We conducted pilot-scale experiments, and found that the improved Faster RCNN met the requirements in terms of detection accuracy and real-time performance during the experimental stage.

## Discussion

The complex detection environment affects the detection accuracy of the improved Faster RCNN to a certain extent. It can be seen from the comparison experiment that the *Auricularia auricula* quality classification model can not detect the target in the image well when the *Auricularia auricula* gets stuck together and under complex lighting conditions. *Auricularia auricula* sticking to each other results in inaccurate identification and classification. The complex lighting conditions environment leads to the inaccuracy detection of *Auricularia auricula* in the image. When preparing the test environment, the separation of the *Auricularia auricula* should be added before *Auricularia auricula* enter the image acquisition area, so that the model can detect a single *Auricularia auricula* as much as possible. To detect a single *Auricularia auricula* as much as possible, the *Auricularia auricula* will be separated before they enter the image acquisition area.

This paper explores the influence of different resolutions on the appearance classification method for *Auricularia auricula*. By comparing the results of images with two different resolutions, images with low resolutions have higher scores and can also obtain accurate coordinates under the condition of multiauricula detection. At the same time, the lower resolution can reduce the input burden of the computer.

The Faster RCNN model can classify the *Auricularia auricula* with an average accuracy of 84.20%, but its real-time performance is poor and can not meet the actual production needs. In order to meet the requirements of accurate appearance quality classification of *Auricularia auricula*, the feature pyramid RPN model is fused with VGG 16 network, and a new multi-scale feature fusion model is constructed by combining the top-down and bottom-up feature fusion methods to enrich the semantics of features at each scale and improve the classification accuracy.

Finally, the appearance quality classification method based on the improved Faster RCNN improves both the classification performance and the real-time performance.

## Conclusions

In order to automatically classify the appearance quality of *Auricularia auricula*, this paper proposes a multiscale feature fusion detection model to improve the standard Faster RCNN. Since the improved Faster RCNN contains more detailed feature information by directly fusing existing shallow feature information from the feature extract network, higher classification accuracy and real-time detection performance are achieved. The appearance quality classification results are compared with the other four deep learning methods. Comparison results show that although the five methods can classify the appearance quality of *Auricularia auricula*, the improved Faster RCNN performs better, with classification accuracy up to 86.33% and FPS up to 13.5 frames/s. These results indicate that the deep learning method based on the improved Faster RCNN could be applied to the appearance quality classification of *Auricularia auricula*.

The study also investigates the influence of image resolution and complex environments, such as *Auricularia auricula* getting stuck together and complex lighting conditions, on deep learning methods. The results reveal that low resolutions yield higher scores and accurate coordinates, particularly in cases of multi-Auricularia auricula detection. However, when *Auricularia auricula* are stuck together or under complex lighting conditions, it negatively impacts the classification results. Therefore, it is recommended to separate the *Auricularia auricula* before they enter the image acquisition area to mitigate these challenges.

One possible reason for the poor performance of algorithms in complex environments is there limited samples for training. Through there are not many scenes where complex environments are encountered under normal working conditions, we will try to collect more samples in these environments for training. We will further improve the appearance quality classification method by studying the possible effects of the number and size of anchor points. Continuing to improve the quality of the training data set will also help greatly in improving the performance of the model. In the process of using this appearance quality classification method, more detection scenarios that may be encountered but not covered by this research will be continuously collected and labeled.

## Data Availability

This dataset has been made publicly available at https://github.com/liyang005/Auricularia_auricula-dateset.
